# Listen-and-repeat training in the learning of non-native consonant duration contrasts: influence of consonant type as reflected by MMN and behavioral methods

**DOI:** 10.1007/s10936-022-09868-6

**Published:** 2022-03-21

**Authors:** Antti Saloranta, Leena Maria Heikkola, Maija S. Peltola

**Affiliations:** 1 Department of Future Technologies, University of Turku, Turku, Finland; 2 Learning, Age & Bilingualism laboratory (LAB-lab), University of Turku, Turku, Finland; 3 Department of Finnish, Åbo Akademi University, Turku, Finland

**Keywords:** Event-related potentials, Mismatch negativity, Production training, Second language acquisition, Speech segmentation

## Abstract

Phonological duration differences in quantity languages can be problematic for second language learners whose native language does not use duration contrastively. Recent studies have found improvement in the processing of non-native vowel duration contrasts with the use of listen-and-repeat training, and the current study explores the efficacy of similar methodology on consonant duration contrasts. 18 adult participants underwent two days of listen-and-repeat training with pseudoword stimuli containing either a sibilant or a stop consonant contrast. The results were examined with psychophysiological event-related potentials (mismatch negativity and P3), behavioral discrimination tests and a production task. The results revealed no training-related effects in the event-related potentials or the production task, but behavioral discrimination performance improved. Furthermore, differences emerged between the processing of the two consonant types. The findings suggest that stop consonants are processed more slowly than the sibilants, and the findings are discussed with regard to possible segmentation difficulties.

## Introduction

The duration of individual segments plays a varied role in the languages of the world. In some languages, duration differences of vowels and consonants are either nonexistent or tied to other features, such as the tense-lax separation in certain variants of English, where vowel duration changes along with vowel quality. In these situations, duration alone does not affect the meanings of words. In so called quantity languages, however, such as Finnish, Estonian, Hungarian and Japanese, variation of segment duration can occur in various positions in a word and be phonologically distinctive. These types of distinctions can be problematic for those second language learners who speak languages where duration contrasts are less significant or do not exist, both for vowels (e.g. McAllister et al., 2002; Tsukada et al., 2014) and consonants (e.g. Hayes-Harb 2005; Porretta & Tucker, 2015). Learning issues such as this are typically tackled with laboratory training, and promising learning results have been achieved with other non-native contrasts, particularly using identification and discrimination training (e.g. Bradlow et al., 1997; Iverson & Evans, 2009; Logan et al., 1991; Strange & Dittmann, 1984). Several studies have indeed reported some success in training duration contrasts with behavioral training methods, most commonly identification training (e.g. Hirata 2004; Hirata et al., 2007; Okuno, 2014; Tajima et al., 2008) and audiovisual training (Motohashi-Saigo & Hardison, 2009). These duration studies used adult or young adult participants, and most, though not all, reported generalization to either novel talkers, untrained stimuli or both. Improvement in identification performance has been observed both for vowels and for consonants, and two of the aforementioned studies saw improvements in the production of duration contrasts in vowels (Okuno, 2014) and consonants (Motohashi-Saigo & Hardison, 2009) as a result of identification training with no additional production component.

A method that is largely unused in training studies is production training, in spite of recent results showing improved performance for several second language features, for example vowel quality (Jähi et al., 2015; Saloranta et al., 2015; Taimi et al., 2014), vowel duration (Saloranta et al., 2020, 2017), and voice onset time (Tamminen et al., 2015; Tamminen and Peltola, 2015). Both Jähi et al. (2015) and Taimi et al., (2014) used listen-and-repeat training to improve the production of a novel vowel quality contrast with linguistically oriented seniors and 7-10-year-old children, respectively. The training consisted of 120 repetitions of the contrast in four sessions over two days. Both groups were able to alter their production of the novel contrast by the second day of the experiment. Saloranta et al. (2015) used a slightly modified version of the same training paradigm with 18-30-year-old adults. The training was enhanced with instructions aimed at making the participants explicitly aware of the feature being trained, resulting in them modifying their production already after one training session. Saloranta et al., (2017) again used a two-day listen-and-repeat paradigm with 120 repetitions of a vowel duration contrast. The participants, who did not have phonological duration contrasts in their native languages, trained using a semisynthetic pseudoword pair, differing in the duration of the first syllable vowel. Their performance was also measured on an untrained pair with a different vowel. The training resulted in improved behavioral discrimination scores and more native-like long/short syllable ratios in the production of the trained stimuli, with no generalization to an untrained stimulus pair or a non-linguistic sinusoidal tone pair. Finally, Tamminen & Peltola (2015) and Tamminen et al. (2015) used a listen-and-repeat paradigm to train a voice onset time (VOT) contrast (/fi:l – vi:l/) found in English, but not in Finnish, to 18-32-year-old native Finnish speakers. Both studies used 120 repetitions of the target contrast during training over three days, resulting in increased accuracy scores and decreased reaction times in behavioral discrimination tasks, changes in category boundary steepness in behavioral identification tasks, together with a shift in the category boundary in the former study and improved stimulus goodness ratings in the latter. The studies also found psychophysiological learning effects using electroencephalography (EEG).

Psychophysiological learning effects are typically examined with event-related potentials (ERP), more specifically the mismatch negativity (MMN). The P3 response is used to indicate plastic changes. Mismatch negativity is a fronto-centrally distributed event-related potential (Näätänen et al., 1997). It has been shown that MMN is elicited when a tone changes in frequency, duration, or intensity (Näätänen & Escera, 2000), occurring even when participants are not attending to the stimuli (Kujala et al., 2007). Most MMN studies use the so called ’oddball’ paradigm, where a stream of identical “standard” stimuli is presented to the listener, occasionally interspersed by “deviant” stimuli that differ from the standards in a specific way. If the listener’s brain can distinguish the deviant stimulus, it elicits an MMN response between 120 and 250 ms (Steinhauer, 2014). As the response can be elicited in the absence of conscious attention, it does not rely on a behavioral response and it has been interpreted to reflect pre-attentive processes (for a review, see Näätänen 2001). The MMN has been shown to be language specific, as the MMN amplitude elicited by the same phonetic contrast varies depending on whether or not the contrast is phonologically relevant in the participant’s native language (Näätänen et al., 1997). It has been suggested that the language specific MMN is most likely based on the formation of permanent memory traces for native language phonemes within the first year of life (Cheour et al., 1998). It has also been shown (Shestakova et al., 2003) that the acquisition of appropriate distinctions between L2 phonemes is crucial for language learners. An MMN response to separate non-native phonemes has been shown for both early L2 (Shestakova et al., 2003), and late L2 learners (Winkler et al., 1999).

MMN responses can also be affected by training. Perceptual training, for example, has been shown to enhance MMN responses to consonant voicing contrasts (Tremblay et al., 1997) and to increase MMN amplitude for discrimination of Japanese mora structures (Menning et al., 2002). Of particular interest are studies where listen-and-repeat training was shown to enhance neural responses: Tamminen et al. (2015) were able to elicit an MMN response to a non-native VOT contrast, and Tamminen and Peltola (2015) found an increase in the amplitude of an existing MMN response to the same voicing contrast. Both studies used three days of listen-and-repeat training with Finnish adult learners of English. Finally, Saloranta et al. (2020) were able to increase the MMN amplitude to a Finnish vowel duration contrast with two days of listen-and-repeat training in 19-29-year-old learners of Finnish. Participants, who had no phonological duration contrasts in their native languages, trained using a semisynthetic pseudoword pair, differing in the duration of the first syllable vowel. Their performance was also measured on an untrained pair with a different vowel. The training was successful in enhancing the MMN amplitude for the trained stimulus, but the effect did not generalize to the MMN for the untrained pair. It did, however, affect their N1 responses, suggesting changes in their neural processing of duration at an earlier, non-linguistic level.

P3 is a parietally distributed ERP component elicited by a person’s reaction to a stimulus (Picton, 1992). The P3 occurs in response to rare and relevant events 250–500 ms after the presentation of a stimulus and appears to be associated with stimulus classification and updating in short-term memory. It has been suggested that P3 latency is a function of the time necessary to classify a stimulus on any task-relevant dimension (Kok, 2001; Kutas & Van Petten, 1988), and, like MMN, it has often been reported in studies using an oddball paradigm (Koerner et al., 2017). In a review of P3, Kok (2001) suggests that it is the attention to stimulus processing (that is “task emphasis”) that increases the P3 amplitude, while concurrent working-memory load (that is “dual-task performance”) reduces it. Elicitation of the P3 response is thought to mark an automatic, involuntary shift of attention, particularly to “novel” stimuli (Escera & Corral, 2007), and it has been shown that the more unexpected the stimulus, the larger the P3 amplitude, both for visual and auditory stimuli (Sutton et al., 1965, 1967). An example of this attention shifting by novel stimuli can be found in Ylinen et al., (2016), who found a P3 response, following an MMN response, to deviant stimuli that break native vowel harmony rules. P3 was not elicited by a mere change in vowel quality that did not break vowel harmony.

The purpose of the current study is to answer two main questions: first, can the perception and production of differences in consonant duration be improved with listen-and-repeat training in a similar timeframe as the perception and production of vowel duration, vowel quality or consonant voicing contrasts? Earlier studies have shown some improvement in each of these three contrast types both neurally and behaviorally, but it is not obvious that vowels and consonants should behave identically. Second, if learning occurs, is there a difference between consonant types, more specifically sibilants and stops? The mechanism for increasing segment duration is the opposite in these sounds: for sibilants, it is a more sustained frication noise, while for stops the duration of the silent occlusion period is increased. Second language learners of Japanese, which has phonological vowel and consonant duration contrasts, have shown differences in their identification ability of Japanese stop and sibilant duration differences (Hardison & Motohashi-Saigo, 2010). These types of processing difficulties may affect the learning results with listen-and-repeat training.

## Materials and methods

### Participants

A total of 18 participants (14 female) took part in the study (Table 1). The participants’ hearing was tested on 100–4000 Hz range at 5–25 dB using a Grason-Stadler GSI 18 audiometer and their handedness was evaluated using the Edinburgh Handedness Inventory (Oldfield, 1971). All participants had normal hearing and were right-handed. Prior to their participation, all participants were screened for their eligibility, including age, native language, other spoken languages and neurological conditions or medications. The pre-screening was vital for the study, as it was important to ensure that the participants’ native languages did not contain phonological duration differences in consonant sounds. Participants with native languages containing these contrasts were ruled out in order to ensure similar baseline discrimination skills at the beginning of the experiment. The participants self-evaluated their non-native language skills regarding proficiency level, usage, and exposure. The students had knowledge of 2–8 languages including their native language (M = 4.6, SD = 1.5). The experiment was conducted in English. All expect one participant were recruited among participants on two intensive Finnish language and culture courses in Finland. One of the participants was an exchange student in the same city. The participants volunteered to participate in the study and received a small non-monetary compensation for their participation. All participants gave their written content to take part in the study, and for the use of the data in this and future studies. The study was approved by the Ethics Committee of the University of Turku. Two participants were excluded from EEG analysis due to electrode malfunction, and a further four due to excessive alpha wave noise, but they were included in discrimination and production analyses. This resulted in 12 analyzed participants (9 female, mean age 21.9, mean length of Finnish studies 10.4 months) in the EEG analysis, and the full 18 in the discrimination and production tasks.

**Table 1:** Basic participant information

Total number of participants	18
Mean age, years (stdev)	21.6 (2.7)
Gender	14 F, 4 M
Mean length of Finnish studies, months (stdev)	10.4 (2.4)
Native languages (number of speakers)	Russian (6), German (4), Spanish (2), Czech (1), Lithuanian (1), English (1), Latvian (1), Georgian (1), French (1)
Non-native languages (mean self-evaluated skill score)	Finnish 18 (1.2), English 17 (3.2), Swedish 3 (1.7), German 7 (2), French 4 (1.8), Spanish 4 (1.8), Russian 3 (2.7), Dutch 1 (1.0), Latvian 1 (1.0), Ukrainian 3 (2.3), Lithuanian 1 (1.0)

The self−evaluated language skill score is on a scale of 0 to 4 (1 = basic, 2 = satisfactory, 3 = manages in everyday situations, 4 = excellent)

## Stimuli

The target consonants for the study were the stop consonant /t/ and the sibilant /s/. The phonemes /s/ and /t/ were chosen as both are common phonemes in Finnish and share an alveolar place of articulation, but differ in manner. Furthermore, they can appear in very similar acoustic environments as both singletons and geminates, as shown by the minimal quartet */kansa/ - /kans:a/ - /kanta /– /kant:a/* (English: *population* – *with* – *viewpoint* – *lid*, partitive form). These factors made it possible to examine only the effect of the manner on possible learning results. The consonants were presented embedded into two pseudoword pairs, /tete/-/tet:e/ and /tese/-/tes:e/. The stimuli were created from natural speech produced by a 31-year-old male native Finnish speaker. The /tes:e/ token was recorded first, and its frication period was then adjusted to the desired duration for the short member of the pair, /tese/ using the duration manipulation function in Praat (version 6.036; Boersma & van Heuven 2001). Then, /tet:e/ was created by replacing the frication in /tes:e/ with silence, leaving only the formant transitions to and from it. This resulted in a /t/ sound with a duration identical to the original /s:/ in /tes:e/. Finally, the duration of the silence was matched to the duration of frication for /tese/, creating /tete/. The purpose of this method was to create stimuli that consisted of natural speech, but were still exactly acoustically identical in the parts irrelevant to the contrast being trained. The first 170 ms of all stimuli are exactly identical, as the difference between the consonants does not start until at that point. The exact lengths were decided by native listener assessment by both trained phoneticians and amateur listeners, who also assessed the final modified and judged that they were good matches for the Finnish /s/ and /t/ categories. The reason the stimuli were created in this way was due to the fact that consonant quantity contrasts in Finnish are typically not accompanied by significant changes in voicing or other quality-related features; in fact, the prevailing phonological interpretation of geminate consonants is that they consist of a sequence of two identical short phonemes (Suomi et al., 2008, pp. 39–40). Therefore, simply shortening the frication and occlusion periods for /s/ and /t/, respectively, is enough to create realistic duration contrasts in the stimuli.

**Fig. 1:**
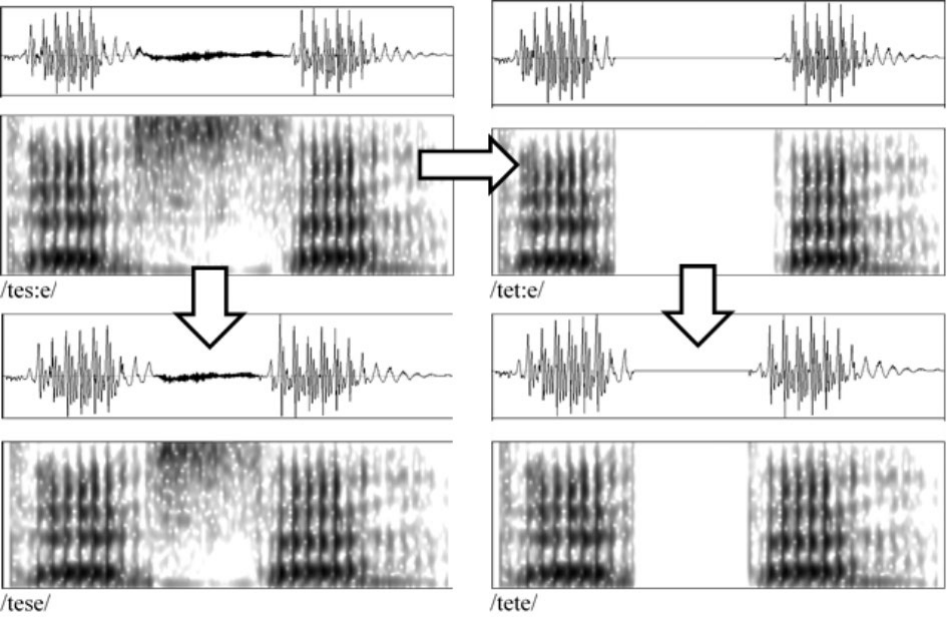
Illustration of the stimulus creation process and spectrograms of the stimulus files. The arrows indicate modification: /tes:e/, the original recording, was modified to create /tese/ and /tet:e/, and /tet:e/ was modified to create /tete/

Spectrograms and waveforms of the stimuli can be seen in Fig. 1. The long member of both pairs was 347 ms long (target sound 119 ms), and the short member 301 ms (target 73 ms), resulting in a difference of 46 ms. The F1 and F2 formant values of the vowels were 479 and 1663 Hz for the first vowel, and 530 and 1573 Hz for the second. Mean fundamental frequency for the voiced sections of the stimuli was 110 Hz. The stimuli were recorded and edited mainly with Audacity (version 2.2.2); the editing of frication length was done in Praat.

## EEG recordings

A Brain Products actiCHamp system and Brain Products Recorder software (version 1.20.0801) was used for the EEG recordings. The system consisted of 32 active electrodes for the EEG and two passive electrodes above and below the left eye to record vertical eye movement; horizontal eye movement was recorded with electrodes F7 and F8 at the sides of the head. The impedance of the electrodes was kept below 10 kΩ. Participants sat still watching a film with no sound while the stimuli were presented binaurally through Sennheiser HD 25 − 1 II headphones with a PC running NeuroBehavioral Systems’ Presentation (version 16.3). An oddball paradigm with a deviant probability of 0.13 (874 standards, 140 deviants) and an interstimulus interval of 650 ms was used to present the stimuli with the short members of the pairs as the standards and the long ones as the deviants. The order of presentation was pseudorandom, with no less than three standards between each deviant.

**Fig. 2:**
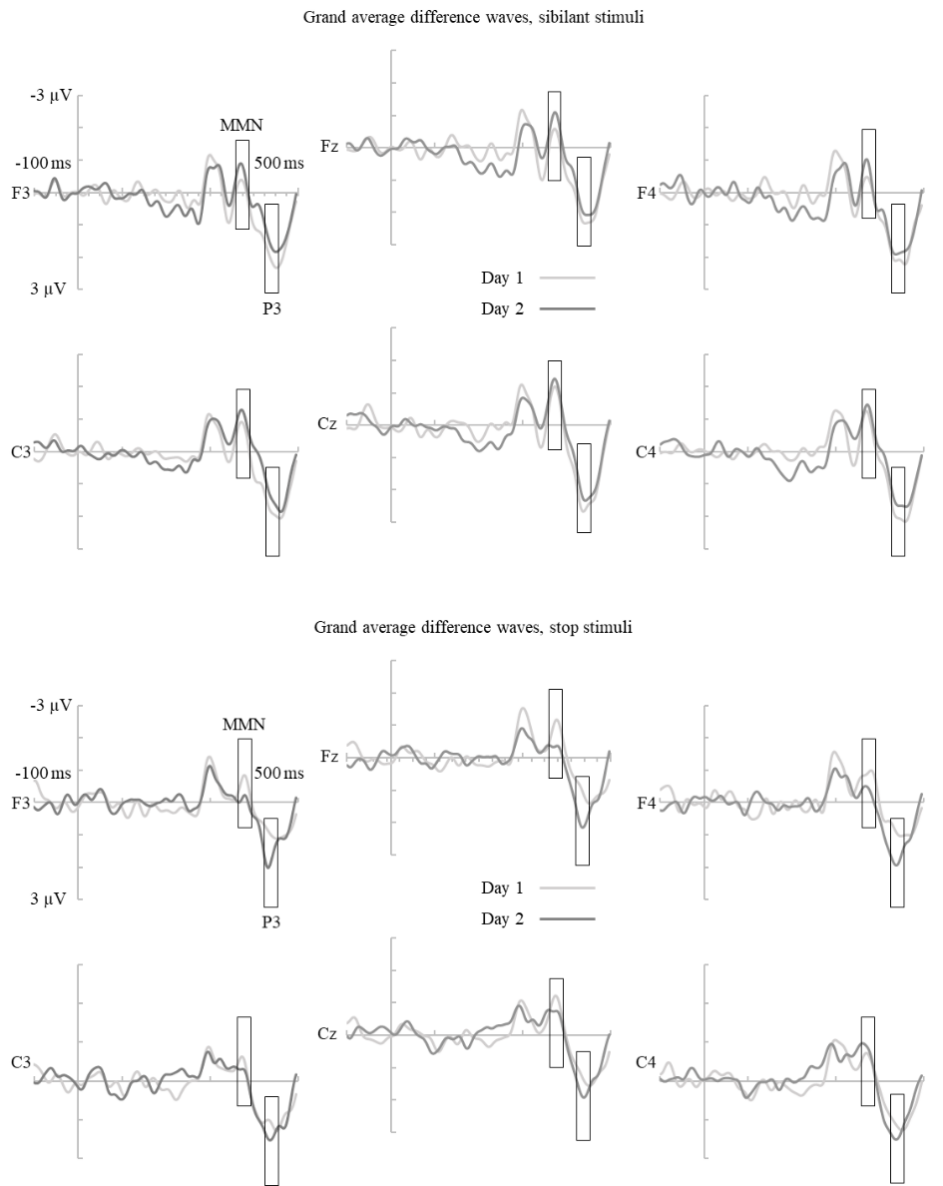
Grand average difference waveforms for the sibilant and stop stimuli for the C3, C4, Cz, F3, F4 and Fz electrodes. Boxes indicate the time windows for each ERP. NB: the difference between the standard and deviant stimuli begins at 170 ms for both stimulus pairs

For analysis, the EEG was offline filtered with a 1–30 Hz bandpass filter and referenced to the average of the left and right mastoids. Artifact rejection was set at ± 100 µV. Analysis epochs started 100 ms before stimulus onset and ended 500 ms after it. Baseline correction was performed using the 270 ms period consisting of the 100 ms prestimulus phase and the 170 ms phase before change onset that is identical in both members of the stimulus pair. The first two standard stimuli following every deviant were excluded from analysis. Separate averages were calculated for all valid standard and deviant epochs, and difference waveforms were then acquired by subtracting the standard waveforms from the deviants. 30 ms time windows for statistical analysis were then chosen. The same time windows, centered around the peak amplitudes in the difference waveforms, were used for both the stop and the sibilant stimuli, as the peak amplitudes occurred at roughly the same time in both stimuli on both days. Two clear negative peaks could be observed in the difference waveforms (Fig. 2). However, given that the difference between the stimuli only started at 170 ms, the time range of the first peak suggests that it is the N1, which is not among the responses examined in this study. The MMN window was therefore set at 360–390 ms, around the second peak, and the P3 window at 425–455 ms. Mean amplitudes were analyzed for each window and used in statistical analyses from electrodes C3, C4, Cz, F3, F4 and Fz.

## Discrimination task

An oddball paradigm with an interstimulus interval of 1000 ms was used in the discrimination task, with the short members of the pairs acting as the standard stimuli and the long ones as the deviants. The deviants were presented with a probability of 0.13 (130 standards, 20 deviants) with the same PC as the EEG stimuli. Participants were instructed to press a button as fast as they could when they heard a deviant stimulus. The number of correct responses, misses, “false alarm” responses and correct rejections was recorded, and they were used to calculate a discrimination accuracy (*d’*) score that was used in statistical analysis of the results; ceiling level is 4.62, while no correct responses earns a score of 0.7. The formula used was d’ = z(H) - z(F), where H is the hit rate, calculated by dividing the number of hits by the number of deviants, and F is the false alarm rate, calculated by dividing the number of false alarms by the number of standards. If the number of hits or false alarms was zero, the value 0.5 was used in order to avoid the d’ value becoming infinite (MacMillan & Creelman, 2005, pp. 6–9). Reaction times to the deviants were also recorded and the average reaction times were used in statistical analyses.

## Production task and training

Both the production task and the training followed the same basic listen-and-repeat structure, where the short and long members of the stimulus pairs were played to the participants in alternating pattern with an interstimulus interval of 3000 ms using the Sanako SLH-07 headset and Sanako Lab 100 language lab software and hardware. Participants were instructed to carefully listen to and repeat each token as accurately as they could. In the production task, the stimulus pairs were presented 10 times, and in the training blocks 30 times for a total of 140 repetition of both the sibilant and stop stimulus pairs (120 during training and 20 during production task). No feedback was given either during the training or the production task. Recordings from the production tasks were acoustically analyzed to determine the average durations of the target consonants in the utterances of each participant, and these were then used to calculate long/short ratios by dividing the duration of the supposedly long segment with duration of the supposedly short one. This relative measurement was used to normalize differences in speaking rate between participants. A ratio of 1.2, for example, would indicate that the participant’s repetition of the long consonant was on average 20% longer than the short one. These ratios were used in statistical analyses. All statistical analyses were performed with IBM SPSS Statistics 22.

## Test structure

A two-day structure was used in the study. Baselines were measured at the beginning of the first day, starting with the EEG recordings, then the discrimination tasks and then the production tasks. These were followed by the first four training blocks (two blocks for both the sibilant and the stop stimuli). The second day was performed in fully reversed order, starting with the final four training blocks, and followed by the production task, discrimination tasks and finally the EEG recordings. The order of the tasks was counterbalanced so that one half of the participants performed every task and training block first with the sibilant stimuli and then the stop stimuli, and vice versa for the other half. Participants were able to take breaks between each task.

## Results

### MMN

Statistical analysis of the EEG recordings begun with one-sample t-tests on the mean amplitudes of the MMN responses in the Fz and Cz electrodes in order to determine whether the responses differed from zero. This revealed that the MMN response was significant for both the sibilant stimuli, apart from the Fz electrode in the pre-test, and the stops, apart from the Fz electrode at post-test (Table 2). The analyses were continued with a Word(2) X Session (2) X Electrode (6) repeated measures ANOVA, including both stimuli at pre- and post-test, in order to determine whether there had been any training effects or differences between the words. This analysis resulted in a significant effect of Electrode (*F*(5,55) = 6.350; *p* < 0.001; η_p_^2^ = 0.366) and a Word X Electrode interaction (*F*(5,55) = 3.993; *p* = 0.004; η_p_^2^ = 0.266), suggesting differences in the mean amplitudes between the electrode sites between stimulus types, and a significant Word X Session X Electrode interaction (*F*(5,55) = 4.999; *p =* 0.001; η_p_^2^ = 0.312). Analysis was then continued with a Word(2) X Electrode(6) repeated measures ANOVA with both stimulus pairs at pre-test in order to determine the cause for the electrode interactions. This resulted in a significant main effect of Electrode (*F*(5,55) = 3.782; *p* = 0.005; η_p_^2^ = 0.256) and a Word X Electrode interaction (*F*(5,55) = 7.153; *p <* 0.001; η_p_^2^ = 0.394). The same analysis for the post-test resulted in a significant main effect of Electrode (*F*(5,55) = 6.251; *p <* 0.001; η_p_^2^ = 0.362). Paired samples t-tests were carried out between the words for each electrode within the same sessions (i.e. C3 electrode for the sibilants against the C3 electrode for stops at pre-test, then C4 for the same etc.). None of these tests reached significance. Further analysis was carried out by analyzing the pre- and post-test differences with a Session(2) X Electrode(6) repeated measures ANOVA with each stimulus pair separately. For the sibilant stimuli, this resulted in a significant main effect of Electrode (*F*(5,55) = 2.169; *p <* 0.001; η_p_^2^ = 0.339). For the stops, a significant main effect of Electrode (*F*(5,55) = 4.852; *p =* 0.001; η_p_^2^ = 0.306) and significant Session X Electrode interaction (*F*(5,55) = 4.044; *p =* 0.003; η_p_^2^ = 0.269) were found. This suggests that the MMN responses to the sibilants did not undergo any training-related changes, but some electrodes differed between pre- and post-test for the stops. Finally, paired samples t-tests were carried out with both words for each electrode between pre- and post-test, but none of these reached significance.

**Table 2:** Average EEG amplitudes

Fz	sibilant		stop	
	MMN	P3	MMN	P3
Pre-test	-0.29 (1.19)	2.20 (1.17)	-0.91 (0.95)*	1.13 (0.72)
Post-test	-0.75 (1.07)*	1.92 (1.40)	-0.32 (0.85)	1.93 (1.23)
Cz	sibilant		stop	
	MMN	P3	MMN	P3
Pre-test	-0.89 (1.27)*	2.46 (1.30)	-0.97 (0.85)*	1.34 (0.85)
Post-test	-1.11 (1.04)*	2.11 (1.49)	-0.67 (0.76)*	1.74 (0.96)
C3	sibilant		stop	
	MMN	P3	MMN	P3
Pre-test	-0.64 (1.15)	1.75 (0.97)	-0.40 (0.69)	1.12 (0.64)
Post-test	-1.02 (1.01)	1.31 (0.99)	-0.23 (0.64)	1.40 (0.88)
C4	sibilant		stop	
	MMN	P3	MMN	P3
Pre-test	-0.90 (1.03)	1.89 (1.10)	-0.56 (0.69)	1.11 (0.77)
Post-test	-1.13 (1.05)	1.49 (1.18)	-0.72 (0.68)	1.40 (0.76)
F3	sibilant		stop	
	MMN	P3	MMN	P3
Pre-test	-0.15 (1.23)	1.98 (1.02)	-0.56 (0.96)	0.96 (0.84)
Post-test	-0.55 (1.00)	1.54 (0.98)	-0.09 (0.90)	1.69 (0.91)
F4	sibilant		stop	
	MMN	P3	MMN	P3
Pre-test	-0.24 (0.94)	2.04 (1.18)	-0.89 (0.92)	0.88 (0.67)
Post-test	-0.57 (1.01)	1.84 (1.13)	-0.34 (0.92)	1.77 (0.89)

Average EEG amplitudes (µV) and their standard deviations (in brackets) for each stimulus type for the electrodes Cz, Fz, C3, C4, F3 and F4. Responses that differ significantly from zero are marked with an asterisk (only analyzed for Cz and Fz).

### P3

One-sample t-tests of the P3 responses were conducted with the Cz and Fz electrodes in order to determine whether they differed from zero. This revealed that the response differed significantly for both stimulus types at both pre- and post-test (Table 2). In order to locate potential training effects or differences between the words, a Word(2) X Session (2) X Electrode (6) repeated measures ANOVA was then conducted. A significant main effect of Electrode (*F*(5,55) = 7.725; *p <* 0.001; η_p_^2^ = 0.413) was found, suggesting overall differences between the amplitudes of different electrode sites. No other main effects or interactions reached significance, suggesting no training-related changes or differences between stimulus types.

## Discrimination task

**Fig. 3:**
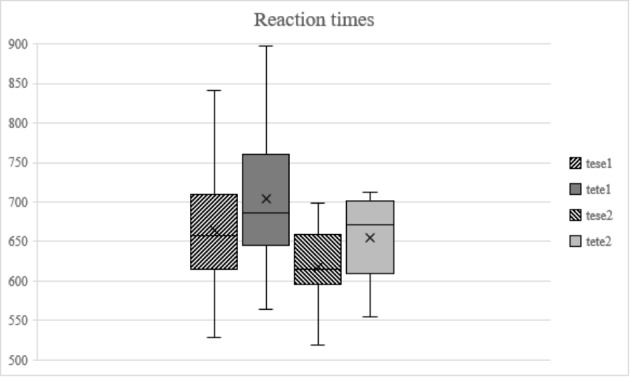
Average discrimination reaction times in milliseconds (Y-axis) for the sibilant (tese) and stop (tete) stimuli on each day (1 = pre-test, 2 = post-test). The box represents approximately 50% of the participants, and the whiskers mark upper and lower ranges. The horizontal line marks the median value

Statistical analysis of the discrimination task reaction times (Fig. 3) began with a Session (2) X Word (2) repeated measures ANOVA. This resulted in the main effects of Session (*F*(1, 17) = 11.004; *p* = 0.004) and Word (*F*(1, 17) = 9.238; *p* = 0.007), suggesting that the pre- and post-test had different reaction times, and that reaction times were different between the two words. In order to examine these effects more closely, paired sample t-tests were then run, first comparing the reaction times of each word between pre- and post-test. The differences were significant for both stimulus types: *t*(17) = 3.260; *p* = 0.005; *d* = 0.626 for the sibilants and *t*(17) = 2.519; *p* = 0.022; *d* = 0.660 for the stops, revealing that the post-test reaction times were faster for both stimulus types. Then, another paired samples t-test was run, comparing the two stimulus types within the same session. Here, the difference was not significant at pre-test, but was so at post-test (*t*(17) = -3.377; *p* = 0.004; *d* = 0.747), suggesting that the reaction times for the sibilant stimuli were significantly faster than the times for the stop stimuli on the second day.

**Fig. 4:**
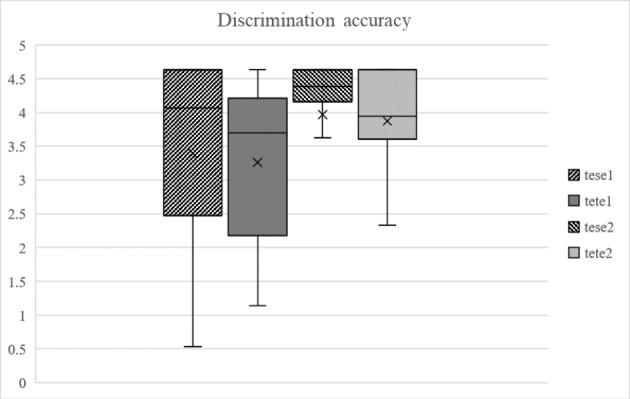
Average discrimination accuracy scores (Y-axis) for the sibilant (tese) and stop (tete) stimuli on each day (1 = pre-test, 2 = post-test). The box represents approximately 50% of the participants, and the whiskers mark upper and lower ranges. The horizontal line marks the median value

Discrimination accuracy (Fig. 4) was then examined statistically, starting with a Session (2) X Word (2) repeated measures ANOVA. This resulted in the main effect of Session (*F*(1,17) = 8.725; *p =* 0.009; η_p_^2^ = 0.339), suggesting changes in overall discrimination accuracy between pre-test and post-test. In order to find out the cause of the effect, paired samples t-tests were run, comparing discrimination accuracy scores for each stimulus type between pre-test and post-test. These resulted in statistically significant differences for both the sibilants (*t*(17) = -2.709; *p =* 0.015; *d* = 0.426) and the stops (*t*(17) = -2.738; *p =* 0.014; *d* = 0.607), showing that the training indeed resulted in an overall increase in discrimination accuracy, although no difference was found between the stimulus types.

## Production task

**Fig. 5:**
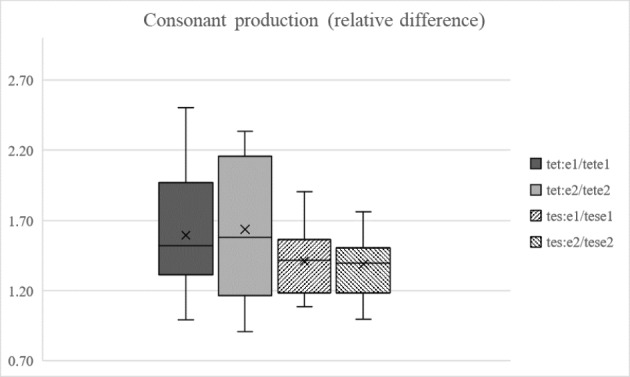
Average long/short ratios (Y-axis) for the consonants of the sibilant (tese/tesse) and stop (tete/tette) stimuli on both days (1 = pre-test, 2 = post-test). The box represents approximately 50% of the participants, and the whiskers mark upper and lower ranges. The horizontal line marks the median value

Statistical analysis of the production data started with a Session (2) X Word (2) repeated measures ANOVA of the long/short ratios of the target consonants. This resulted in a main effect of Word (*F*(1,17) = 15.459; *p = <* 0.001; η_p_^2^ = 0.476), indicating overall differences in the ratios between the stimulus types. Paired samples t-tests were then performed, comparing the ratios between the stimulus types in the same sessions. The difference was significant in both the first (*t*(17) *=* 2.543; *p =* 0.021; *d* = 0.547 ) and second (*t*(17) = 3.096; *p* = 0.007; *d* = 0.704) sessions, showing that the participants produced the long and short stops with a significantly larger difference than the sibilants throughout the experiment (Fig. 5). No training-related effects of any kind were found for the production ratios.

## Discussion

The aim of the study was to answer two main research questions. First, can the perception and production of differences in consonant duration be improved with listen-and-repeat training in a similar timeframe as the perception and production of vowel duration, vowel quality or consonant voicing contrasts? Second, if learning occurs, is there a difference between consonant types, more specifically sibilants and stops? Listen-and-repeat training has been shown to improve both the perception and production of vowel duration in a short time (XXXXXXXX). Thus, it was expected that the protocol used in this study would lead to improvements in the perception and production of consonant duration oppositions as well. However, mixed results were obtained. The psychophysiological measurements were inconclusive with no clear statistical evidence for learning effects. On the other hand, the behavioral discrimination tests revealed improvement between pretest and posttest in both discrimination accuracy and reaction times, as well as a difference between the stimulus types in the lower reaction times for the sibilants at posttest. While no training-related improvement was found in the production results, there was a difference between the stimulus types, as the stops were produced with a significantly longer short-long difference than the sibilants.

The results from the study seem to indicate that perception and production of consonant duration cannot be improved equally well as that of vowel quality (Jähi et al., 2015; Saloranta et al., 2015; Taimi et al., 2014), vowel duration (Saloranta et al., 2020, 2017) or voice onset time (Tamminen et al., 2015; Tamminen and Peltola, 2015) with a similar amount of listen-and-repeat training. No improvement was found in the psychophysiological or the production measurements from pretest to posttest. The MMN results in particular were strikingly different to those achieved by Saloranta et al. (2020) with vowel duration. For the consonants, the mean amplitudes of the responses were largely lower than 1 µV throughout the experiment, and they were not uniformly elicited, as suggested by the few nonsignificant results from the t-tests testing the elicitation (Fig. 2). In Saloranta et al. (2020) higher overall MMN amplitudes were found already at the pretest phase and their elicitation was confirmed throughout the experiment. These results suggest a major difference between the effectiveness of listen-and-repeat for the preattentive perception of vowel and consonant duration, with vowels being much more susceptible to this type of training.

The P3 response was elicited systematically for both stimulus types on both test days. Initial exploration of the EEG data suggested there could have been training-related effects, but none emerged in statistical analyses. While learning effects were not found, the fact that P3 was systematically elicitated suggests that the participants’ attention was involuntarily shifted towards the deviant stimuli of the oddball paradigm. This is commonly observed in oddball MMN designs (Escera & Corral, 2007), where the participants who are instructed to ignore the stimulus train may find it difficult to do so for the entire length of the recording blocks.

While the psychophysiological and production results showed no training effects, significant improvement was achieved in the behavioral discrimination test for both stimulus types in both discrimination accuracy and reaction times. While this result at first seems at odds with the MMN responses, it may be explained by a different focus of attention. While the elicitation of the MMN is preattentive and dependent on the detection of a deviation from a native language memory trace, i.e. a phoneme category, no such limitation technically exists for the behavioral discrimination task which can involve conscious decision making. It is therefore possible that the participants were able to direct their attention on linguistically irrelevant acoustic properties of the stimuli and were able to improve their performance in detecting them.

While the learning effects achieved in this study were somewhat ambiguous, clear differences could be observed between the two stimulus types, as suggested by earlier research where second language learners were not equally able to identify stop and sibilant duration contrasts (Motohashi-Saigo & Hardison, 2009). Statistically significant differences between the sibilants and stops were observed in the discrimination reaction times on the second day, and on the production ratios throughout the experiment. All these differences pointed towards the stop stimuli being more difficult to perceive and produce than the sibilants. Perceptually, the participants were slower to react to the stop stimuli after training, suggesting they needed more time to process them before deciding. In the production task, stops were produced with a significantly larger short/long difference than the sibilants. This may be tied to perceptual difficulties as well: the participants may have needed to produce the stops with an exaggerated difference in order to consider the contrast to be large enough.

A partial explanation for the difference between the stimulus types may also lie in the perceived segmentation of the stimuli. Participants were provided with no contextual information about the words they heard in the study, nor were they given feedback at any stage. The purpose of this was to let them find the relevant difference between the short and long members of the stimulus pairs themselves, similarly to natural speech learning situations. This, however, introduces the possibility for unintended interpretations of the stimuli. Quené (1992) studied the way in which variations in intervocalic consonant duration in connected speech affect the interpretation of word boundaries in Dutch. In the study, the stimulus words were presented in sentences with ambiguous semantic contexts, and the duration of intervocalic consonants was artificially changed, keeping other acoustic information constant. The study found that varying the duration of the consonant significantly affected the listeners’ interpretation of the stimuli, and they concluded that “acoustic-phonetic cues contribute to word segmentation, at least under conditions where no other information is available” (Quené 1992: 345). In our study, where semantically meaningless pseudowords were used as the stimuli, it is impossible to make judgments on word boundaries based on anything other than acoustic information. Depending on the native language of each participant, it may therefore be possible that some of them interpreted the longer members of the stimulus pairs as two different words. This is especially likely for the stop stimuli, as the increase in duration is achieved through a lengthened silence, possibly enhancing the perceived distance between the two syllables, rather than continued frication as in the sibilant stimuli. Furthermore, while single-word interpretations of all the stimuli are semantically meaningless, /tete/ and particularly /tet:e/ could conceivably be interpreted as the Finnish words “te, te” (“you, you”), which may seem like a plausible option to the participants, who are all students of Finnish. In this case, the larger production ratios for the stop stimuli could be explained by some participants attempting to produce two separate short words in sequence, rather than a single one with a central stop consonant. Slower reaction times for the stops in the behavioral discrimination task could also be explained by this interpretation, if at least some of the participants thought the deviant consisted of two words rather than one. The current study design, however, does not allow us to confirm this interpretation and further study would be needed to shed light on the matter.

All in all, the mixed results of the training in this study runs somewhat counter to the results achieved by perceptual training of non-native consonant duration contrasts (Hirata, 2004; Hirata et al., 2007; Okuno, 2014; Tajima et al., 2008) and, indeed, other listen-and-repeat studies with other non-native contrasts (Jähi et al., 2015; Saloranta et al., 2015; Taimi et al., 2014; Tamminen et al., 2015; Tamminen and Peltola, 2015) and non-native duration contrasts (Saloranta et al. 2017, Saloranta et al. 2020). The complete lack of psychophysiological learning effects, in particular, runs counter to Tamminen and Saloranta’s earlier results where two days of listen-and-repeat training was enough to elicit or enhance MMN responses to non-native contrasts. It may be that more training is needed to achieve similar results with consonant duration, and further study should be conducted to determine how much more resistant to improvement this contrast is. It may also be beneficial to present the training stimuli embedded in natural contexts, rather than as individual words, in order to eliminate ambiguous interpretations and assure that the correct acoustic cues are attended to.
